# Cadmium affects toxicokinetics of pyrene in the collembolan *Folsomia candida*

**DOI:** 10.1007/s10646-011-0839-2

**Published:** 2012-02-11

**Authors:** Mieke Broerse, Hilde Oorsprong, Cornelis A. M. van Gestel

**Affiliations:** Department of Animal Ecology, Faculty of Earth and Life Sciences, VU University Amsterdam, De Boelelaan 1085, 1081 HV Amsterdam, The Netherlands

**Keywords:** Uptake and elimination kinetics, Polycyclic aromatic hydrocarbons, Biotransformation, Soil arthropods, Mixtures

## Abstract

Since toxicity is time dependent, short-term toxicity tests may overlook mixture effects, because chemical accumulation within an organism takes time. We therefore studied the effects of cadmium on the toxicokinetics of pyrene and its metabolites in the soil-dwelling collembolan *Folsomia candida* exposed through Lufa 2.2 soil. Single pyrene was rapidly taken up and steady state was reached within the 337-h exposure period. Simultaneous exposure to cadmium significantly decreased the pyrene uptake and elimination rate, resulting in a prolonged half life. Kinetics of the first phase metabolite OH-pyrene was also significantly influenced by cadmium. Cadmium increased the hydroxylation rate of pyrene but slowed down its further metabolization, again resulting in a prolonged half life. We showed that pyrene accumulation and metabolization are significantly influenced by the presence of cadmium. Our results suggest that mixture effects may be dependent on exposure time.

## Introduction

When exposed to chemicals, the extent to which an organism may be affected is determined by several aspects that influence the bioavailability, uptake, internal distribution and eventually the concentration and, hence the effect of the chemical at the site of action within the body. If a chemical is taken up by an organism, exposure time is a very important factor to reach an internal effect concentration. When for a given chemical the uptake is slow, but still exceeds the excretion/detoxification rate, the organism will slowly accumulate the chemical, which eventually may lead to toxic effects. For such chemicals the lower exposure concentrations cause no or little acute or short-term toxicity, but may be harmful upon long-time exposure. When studying the (toxico) kinetics, the organism usually is exposed to a substrate (e.g. water, food, soil) contaminated with only the chemical of interest (see e.g. Van Brummelen and Van Straalen 1996; Díez-Ortiz et al. 2010). In reality organisms however, are usually subjected to a broad variety of different chemicals. Once inside the body, different chemicals may be dealt with differently. This is especially true for organic chemicals and metals. Organic chemicals, like Polycyclic Aromatic Hydrocarbons (PAHs), generally distribute over different tissues based on their chemical properties (e.g. molecular size, lipophilicity) and are eliminated through metabolism (phase 1 and 2) or excretion of either the parent compound or the metabolites (Svendsen et al. 2011). Metals can be essential or non-essential for the organism and biochemical pathways have evolved to regulate body concentration of essential metals. Non-essential metals, however, may use some of these pathways and are also taken up actively (Luoma and Rainbow 2008).

PAHs are present in crude oil and formed as by-product of the combustion of organic materials, and therefore are found throughout the environment. Due to their great binding affinity to soil organic matter these lipophillic chemicals may reach concentrations in soil that are considerably higher than elsewhere in the environment (Van Brummelen and Van Straalen 1996). Pyrene is one of the most abundant of the PAHs and always present in PAH mixtures (Jongeneelen 2001; Levin 1995). Pyrene is considered to be non-carcinogenic and its toxicity is believed to be mainly through non-polar narcosis (Jensen and Sverdrup 2003). In Eukaryotes, detoxification usually occurs in two phases. In the first phase (phase 1) the cytochrome P450 enzyme system introduces a functional group, such as hydroxyl, to the non-polar compound. In phase 2, detoxification enzymes such as glutathione S-transferase attach a large polar water-soluble moiety to phase 1 metabolites to promote excretion and elimination (Brown et al. 2004). Besides detoxification, biotransformation may produce metabolites that are more toxic than the parent compound (Di Giulio et al. 1995; cited by Akkanen and Kukkonen 2003). Thus biotransformation not only enhances the number of potentially toxic compounds, but may also change the mode of toxic action (Droge et al. 2006). Cadmium (Cd) is a widespread environmental pollutant due to anthropogenic activities such as mining and fossil fuel combustion. It is one of the most toxic metals, and negative effects may be found already at relatively low concentrations (Luoma and Rainbow 2008). It is non-degradable and readily accumulates in organisms (Janssen et al. 1991; Crommentuijn et al. 1994). Cd has been classified as class 1 human carcinogen (IARC 1993) due to multifactorial mechanisms, such as accumulation of DNA damage due to inhibition of DNA repair enzymes (Huff et al. 2007; and see also Ragunathan et al. 2010 for references). PAHs and metals usually co-occur in soil and their joint adverse effects in a mixture are usually considered independent within an organism, since they act differently (e.g. Price et al. 2002). However, this is an assumption and nothing is known about possible interactions between these chemicals in soil invertebrates.

In this study we investigated if simultaneous exposure to a non-essential metal (Cd) influences the uptake, metabolism and/or the elimination rate of pyrene in the springtail *Folsomia candida* (Collembola) when exposed to pyrene in Lufa 2.2 soil with and without the presence of cadmium. Collembolans are an integral part of soil ecosystems, playing an important role in the functioning of the soil ecosystem, and are vulnerable to soil contaminants (Fountain and Hopkin 2001).

## Materials and methods

### Test design

To derive uptake and elimination rate constants for both pyrene and OH-pyrene, *F. candida* was exposed to soil contaminated with pyrene (nominal concentration of 40 μg Pyr/g dry soil). To assess the effect of cadmium on pyrene toxicokinetics, animals were also exposed to the mixture of pyrene and cadmium (nominal concentration of 40 μg Pyr/g dry soil + 100 μg Cd/g dry soil). In addition, animals were exposed to single cadmium (nominal concentration of 100 μg Cd/g dry soil). All exposures lasted for 337 h (uptake phase). Pyrene and cadmium concentrations were based on previous studies and never exceeded the 28-day LC_50_ values for pyrene and Cd (e.g. Droge et al. 2006; Sørensen and Holmstrup 2005; Van Gestel and Hensbergen 1997; Van Gestel and Van Diepen 1997). Springtails were sampled at different times during the uptake phase (see Table 1 for sampling times). At the end of the exposure period (*t* = 337 h) all remaining springtails were transferred to clean soil (elimination phase) and detoxification of *F. candida* was followed for 216 h (see Table 1 for sampling times). During the uptake phase and the elimination phase, duplicate test containers were sampled at each sampling time. Animals were analysed for pyrene, OH-pyrene and other metabolites at each sampling, while soils samples were analysed for pyrene and Cd at the start and end of the uptake phase (see Table 1).

**Table 1:** Outline of sampling times (in hours) for the uptake and elimination kinetics study with pyrene in *Folsomia candida* in Lufa 2.2 soil, with and without simultaneous exposure to cadmium

Treatment	Uptake phase										Elimination phase						
Hours:	0	4	8	24	36	48	72	146	241	337	337	344	360	384	408	482	553
Pyrene (± Cd)	X	X	X	X	X	X	X	X	X	X		X	X	X	X	X	X
Cd										X							X
Controls	X							X		X						X	X
Soil analyses	X									X	X						X

The table shows moments (X) at which duplicate soil and animal samples were taken for the different treatments

### Test soils

A natural soil from Germany, LUFA—Speyer soil 2.2 (Lufa 2.2) was used. This sandy soil has a pH(H_2_O )of approximately 5.8 and contains 3.9% organic matter and 5.1% clay. All test soils were prepared by adding deionized water to obtain a soil moisture content of 22% (w/w) (50% of the Water Holding Capacity). After adding water or Cd solution (CdCl_2_^.^H_2_O, Aldrich; >98% A.C.S.), soils were left to equilibrate for 4 weeks before further treatment (at 20 ± 1°C). As pyrene (CAS 129-00-0, Riedel-de Haën; 99 + %) is poorly water-soluble, it was dissolved in acetone (Fluka 99.5% Chemie GmbH) before adding to the soil. All soils, except for the water control, were treated with acetone with or without (solvent control) pyrene, by submerging 10% of the soil, per treatment, with acetone. After 24 h equilibration, the acetone was allowed to evaporate for 2 days in a fume cupboard. Dried soil was mixed in with the remainder of the soil and re-moistened to 22% (w/w) by adding deionized water. For all treatments and controls glass test containers (100 ml) were filled with 20 g wet soil and closed with a lid.

Additionally, duplicate jars for soil analyses contained 40 g wet soil. Pyrene-treated soils were protected from light by covering with aluminum foil prior to the experiment to avoid photochemical transformation of the parent compound.

### Uptake and elimination tests

*F. candida* was cultured in the laboratory at 20 ± 1°C with a 12:12 h light:dark regime, using plastic containers with a moist bottom of plaster of Paris mixed with active coal. Animals were age synchronized by allowing individuals from the culture to lay eggs for 2 days in separate containers. Springtails were fed ad libitum with commercial dried baker’s yeast (Oetker) during synchronization and experiment.

Twenty 30 ± 1 day-old age-synchronized springtails were introduced into each test container at the start of the experiment. Twice a week test containers were opened for aeration and compensation of water loss. To derive kinetics parameters, two test containers per treatment were sampled at each sampling time (Table 1) by adding 33 ml water to a test container, stirring gently and transferring all soil, water and springtails into a glass beaker, repeating this procedure three times. Living springtails came floating to the water surface and were gently transferred to a dry bottom of plaster of Paris to get rid of moist and soil particles before being weighed (Mettler Toledo UMT2, ultramicrobalance; accuracy 0.1 μg). If total weight of all springtails per test container was less than 1 mg, animals from the duplicate test containers were pooled. Springtails were stored at −80°C before further analyses.

### Chemical analysis

Soil moisture content was determined by drying soils for 48 h at 60°C. For pH measurements dried soil samples were shaken with a 0.01 M CaCl_2_ solution for 2 h at 200 rpm, at a solution:soil (w/w) ratio of 5:1. After sedimentation of soil particles, pH was measured using a Consort p907 pH meter.

Total Cd concentrations were determined by digesting dried soil samples in a 2:6:2 mixture of HNO_3_ (65%, Riedel-de Haën), HCl (37%, Baker) and demineralised H_2_O using a microwave (CEM MARS 5). This digest was diluted to 25 ml with demineralized water and analysed by flame Atomic Absorption Spectrophotometry (AAS) (Perkin Elmer AAnalyst 100). The certified reference material ISE-989 (River Clay, Wageningen Evaluating Programs) was used to determine the accuracy of the analytical procedure, which was within performance acceptance limits (<4% deviation).

Measurement of total Cd concentrations in the animals was done by three runs of mini-destruction of freeze-dried springtails in a block heater with HNO_3_ + HNO_4_ (Ultrex 2 (71%) and Ultrex; 7:1). After evaporation to dryness, residues were taken up in 300 μl 0.1 M HNO_3_ and analysed by graphite furnace AAS (Perkin Elmer 5,100). The reference material Dolt-2, certified by the National Research Council of Canada as reference material, was used to determine the accuracy of the analytical procedure, which was within 10% of the certified value.

For determining actual pyrene concentrations in the soil, we followed the method described by Leon Paumen et al. (2008) using Soxhlet extraction followed by high performance liquid chromatography (HPLC). The HPLC consisted of a Vydac RP 18 201TP column with a Vydac 201GD RP-18 guard column (Alltech, Breda, The Netherlands), a Jasco FP-1520 fluorescence detector (Jasco, Essex, UK), and a Gynkotek UVD320s ultraviolet diode-array detector (Gynkotek, Germering, Germany).

To measure pyrene and its metabolites (OH-pyrene and five conjugates), springtails were pottered with a Teflon pestle with Tris buffer (pH 8.7) and carbazole as an internal standard and stored for 30 min. at −80°C. After 5 min. of sonication, 400 units of proteinase K solution was added and the sample was vortexed before being incubated at 38°C for 2 h. Ethanol amended with ascorbic acid was added and samples were centrifuged at 12,000 rpm, after which the debris were left in the Eppendorf tubes while liquid was transferred into brown HPLC vials. Supernatants were measured by HPLC applying an elution gradient as described by Stroomberg et al. (2004) using the same equipment as mentioned for soil analysis and with fluorescence detection at λ_ex/em_ = 346/384 nm.

### Data analyses

Pyrene kinetics in the Collembola were described using a one-compartment model. This model considers the animal as a homogeneous compartment with single uptake and elimination rates (e.g., Janssen et al. 1991; Stroomberg et al. 2004). The model parameters and corresponding standard errors were estimated by fitting the following equations simultaneously using non-linear regression in SPSS15.0:

Uptake kinetics (for t ≤ t_e_):

$$ {\text{Q}}\left( {\text{t}} \right) = {\text{k}}_{ 1} /{\text{k}}_{ 2} \times {\text{C}} \times \left( { 1- {\text{e}}^{{( - {\text{k}}_{ 2} \, \times {\text{t}})}} } \right) $$

And elimination kinetics (for t > t_e_):

$$ {\text{Q}}\left( {\text{t}} \right) = {\text{k}}_{ 1} /{\text{k}}_{ 2} \times {\text{C}} \times \left( { 1- {\text{e}}^{{ - {\text{k}}_{ 2} \, \times {\text{t}}}} } \right) \, - {\text{ k}}_{ 1} /{\text{k}}_{ 2} \times {\text{C}} \times \left( { 1- {\text{e}}^{{ - {\text{k}}_{ 2} \, \times ({\text{t}} - {\text{te}})}} } \right) $$

where: Q(t) = pyrene concentration in the organism at time (t) (μg/g fresh body weight), C = pyrene exposure concentration (μg/g dry soil), k_1_ = uptake rate (g dry soil/g fresh body weight, hour), k_2_ = elimination rate constant (per hour), (t) = time (hour), t_e_ = time at which animals were transferred to clean soil (337 h).

The same model was also fit to model kinetics of OH-pyrene formation and elimination in the animals.

To compare pyrene and OH-pyrene kinetics parameters for exposures with or without Cd a generalized likelihood ratio test was performed (Sokal and Rohlf 1995).

Bioaccumulation factors (BAF) were derived by dividing k_1_ by k_2_. The chemical half lifes of pyrene and OH-pyrene in the springtails were calculated using ln2/k_2_.

All calculations were performed using SPSS version 15.0.

## Results

### Soil moisture contents, pH and chemical concentrations

Test soils had moisture contents between 19.1 and 21.3% and an average pH(CaCl_2_) of 5.22 ± 0.12 (SD; *n* = 48). Actual pyrene concentrations in the soil were lower than nominal ones (27.0–30.2 μg pyrene/g dry soil) while for Cd concentrations this was the opposite (116–130 μg Cd/g dry soil), as shown in Table 2. Pyrene concentration in the soil of the pyrene-only treatment remained fairly constant throughout the uptake phase, while in the treatment with Cd, pyrene concentration showed a decrease of 28% after 337 h (Table 2). To calculate kinetics parameters, the average pyrene concentrations of 26.6 ± 4.5 and 26.0 ± 4.9 μg Pyrene/g dry soil (SD; *n* = 4) were used for the single pyrene and pyrene with Cd exposure, respectively. In the uptake tests with cadmium, average Cd concentration in the test soil was 123 μg/g dry soil.

**Table 2:** Pyrene and cadmium concentrations in Lufa 2.2 soil (in μg/g dry weight) at the start and end of the uptake phase during the toxicokinetic test with *Folsomia candida* in Lufa 2.2 soil (±SD; *n* = 2)

Treatment	Uptake (0 h) (μg/g dry soil)	Uptake (337 h) (μg/g dry soil)
Pyrene	27.0 (7.5)	26.2 (2.1)
Pyrene + Cd	30.2 (*n* = 1)	21.8 (0.9)
Cd	116 (0.65)	130 (5.36)

### Toxicokinetics

Not all springtails survived until the end of the experiment, with mortality being most visible in the Cd treatments both with or without pyrene. Unfortunately, we did not quantify mortality. In the control and single pyrene treatments >90% of the *F. candida* survived until they were sacrificed for measuring internal chemical concentrations.

**Fig. 1:**
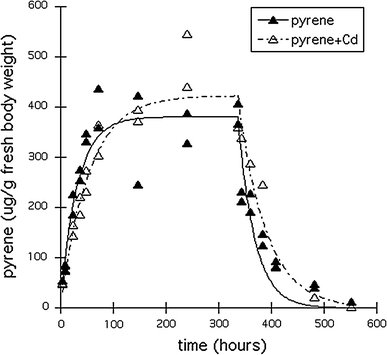
Uptake and elimination of pyrene in *Folsomia candida* exposed to Lufa 2.2 soil treated with approximately 26.5 μg pyrene/g dry soil, with (*open symbols*) or without (*closed symbols*) approximately 123 μg Cd/g dry soil. *Lines* represent fit of the first-order one-compartment kinetics model

**Fig. 2:**
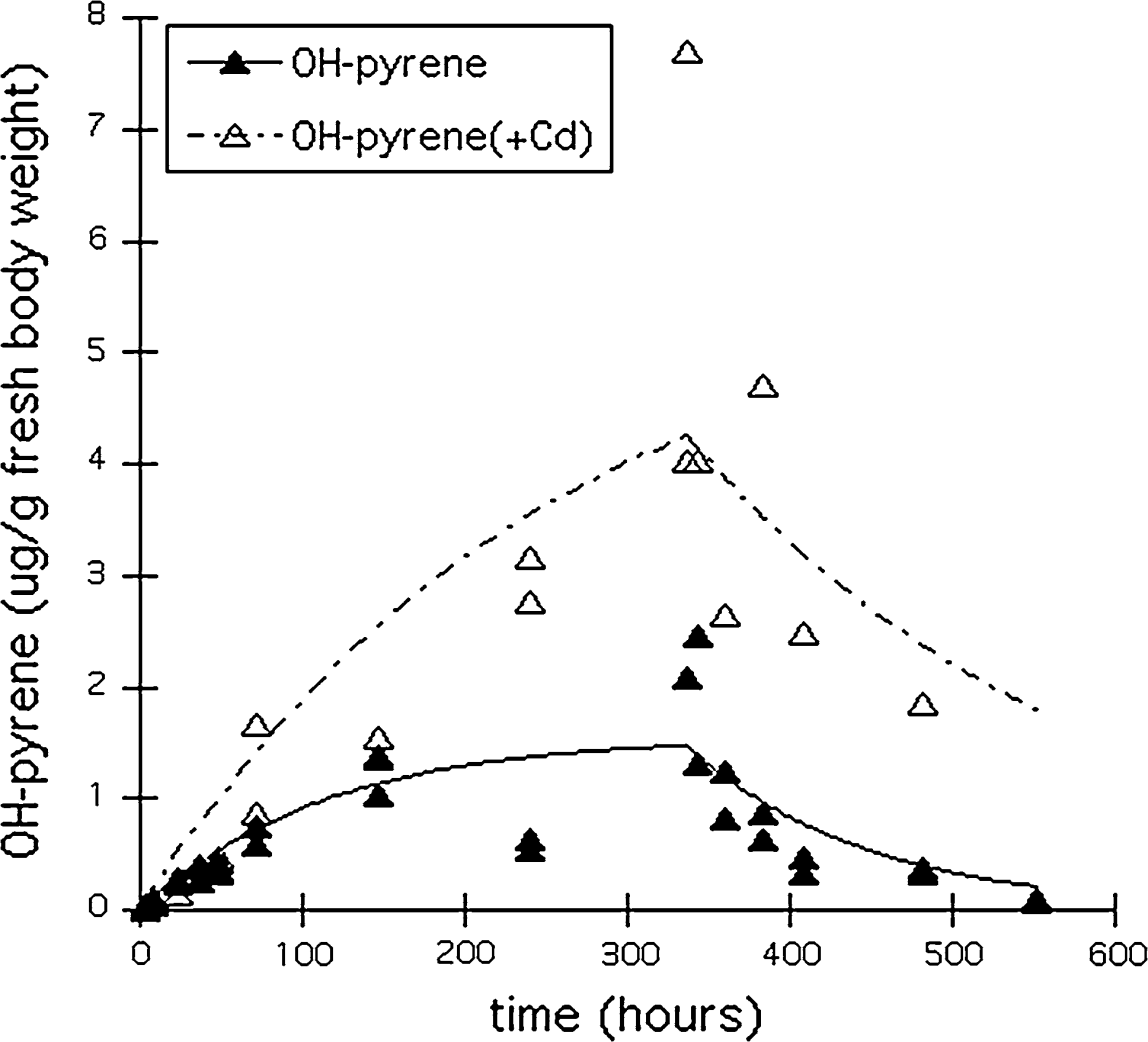
Development in time of internal OH-pyrene concentrations in *Folsomia candida* exposed to pyrene in Lufa 2.2 soil for treatments with (*open symbols*) and without (*closed symbols*) Cd (see Fig. 1 for kinetics of pyrene). *Lines* represent fit of the first-order one-compartment kinetics model

**Fig. 3:**
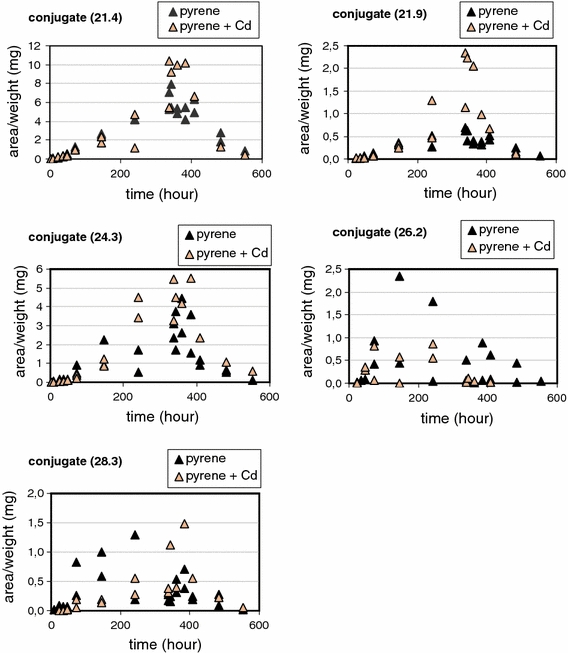
Internal pyrene conjugate concentrations in time in *Folsomia candida* exposed to pyrene in Lufa 2.2 soil for treatments with (*open symbols*) and without (*closed symbols*) Cd (see Fig. 1 for kinetics of pyrene). The different figures relate to different pyrene conjugates; see text for further information. Since no quantification of the concentrations was possible, amounts of the different metabolites are expressed as units (of the HPLC readings) per fresh body weight of the animals

Cd concentrations in the animals did not differ between treatments with and without pyrene and were 59–75 μg Cd/g dry body weight after the 337-h uptake phase and 15–16 μg Cd/g dry body weight at the end of the elimination phase. Figures 1 and 2, respectively show the internal pyrene and OH-pyrene concentrations (μg/g fresh weight) in the Collembola, without or with Cd in the soil. The corresponding toxicokinetics parameters are given in Table 3. Single pyrene was rapidly taken up by the springtails and steady state was well reached within the 337-h uptake period (after approx. 100 h). In the mixture with Cd, pyrene uptake and elimination were significantly slower (likelihood ratio test: X_df_^2^_=1_ > 3.84; *P* < 0.05) (Table 3), and led to more pyrene accumulation in the springtails and a prolonged elimination half life. BAF values were estimated at 14.3 and 16.3 g dry soil/g fresh body weight for exposures without and with cadmium, respectively (Table 3). Pyrene hydroxylation into OH-pyrene was even more influenced by the presence of Cd, resulting in a very long half life (173 h) for OH-pyrene in the springtails. Cd was accumulated in both treatments, i.e. with and without pyrene with on average 67.0 ± 10.9 μg Cd/g dw after 337-h and 15.8 ± 0.45 μg Cd/g dw in the animals at the end of the elimination phase (*t* = 553 h). In addition to OH-pyrene several possible phase 2 metabolites were measured, as shown in Fig. 3. We named them after their retention times and expressed the amounts as area/body weight units, since neither chemical identification nor quantification were done. Internal concentrations of all phase 2 metabolites showed an increase in the uptake phase, followed by a decline during the elimination phase. For two metabolites (21.9 and 28.3), the increasing and decreasing trends were different between exposures with or without Cd. For the other metabolites, trends seemed not affected by cadmium.

**Table 3:** Uptake rate (k_1_) and elimination rate constant (k_2_) (with s.e.) for pyrene and OH-pyrene in *Folsomia candida* following exposure to pyrene in Lufa 2.2 soil with or without cadmium

Treatment	Pyrene	Pyrene + Cd	X^2^; *P*	OH-pyrene	OH-pyrene + Cd	X^2^; *P*
k_1_ (g/g, h)	0.458 (0.046)	0.309 (0.024)	98.7; *P* < 0.001	0.014 (0.003)	0.023 (0.005)	10.0; *P* < 0.01
k_2_ (per h)	0.032 (0.003)	0.019 (0.002)	4.10; *P* < 0.05	0.009 (0.002)	0.004 (0.001)	0.446; n.s
BAF (g/g)	14.3	16.3				
t_1/2_ (h)	21.7	36.5		77	173	

Differences were determined by comparing uptake and elimination kinetics applying generalized likelihood ratio tests (X^2^ at 1 df; n.s. means not significantly different)

## Discussion

We demonstrated that pyrene accumulation and metabolization in the collembolan *F. candida* is significantly influenced by the presence of cadmium.

We estimated an uptake rate for pyrene of 0.458 g/g, hour with an elimination rate constant of 0.032 per hour for single pyrene exposure. It is difficult to compare the pyrene kinetics found in this study with other studies as test organism, exposure medium as well as exposure route differed greatly. The estimated uptake rate differed from those found by Jonsson et al. (2004), exposing sheephead minnows to contaminated seawater. They found that, depending on the exposure concentration, the uptake rate was 5.375 l/g, hour (low) and 4.83 l/g, hour (high). Both uptake rates are much faster than for *F. candida*, which is not surprising since the units are very different. Elimination rate constants (0.037 and 0.049 per hour, respectively) did not differ much from the values found in our study. This suggests that the uptake route through water is different than for soil, but internal elimination might follow the same pathway in some organisms, corresponding with a similar excretion rate. This similarity in elimination rate constants was also found for the elimination of benzo(a)pyrene in *Porcellio scaber* of 0.046 per hour (Van Brummelen and Van Straalen 1996) and for pyrene in *Eisenia andrei* of 0.032 per hour (Jager et al. 2000).

We found a significant influence on pyrene kinetics in the presence of Cd with more pyrene being accumulated and a prolonged half life. In this study we aimed at minimizing toxic effects by using sublethal concentrations. Nevertheless, we observed increased mortality in the treatments with cadmium. Even though the measured cadmium concentration in soil was somewhat higher than anticipated, it did not exceed 28-day LC_50_ values found in other studies (e.g. Sørensen and Holmstrup 2005; Van Gestel and Hensbergen 1997; Van Gestel and Van Diepen 1997) with Askov (1.6% organic carbon) and artificial soils (variable organic matter contents). Also, internal Cd concentrations in the animals did not exceed the internal EC_50_ for the effect on reproduction of *F. candida* reported by Van Gestel and Mol (2003) after 4 weeks of exposure. It therefore remains unclear why mortality was increased in the treatments with cadmium.

Our results showed an interaction between pyrene and Cd that may have consequences for their combined toxicity. There is a lack of information about pyrene uptake and elimination rate constants in the presence of Cd in literature. In addition, the literature data on combined effects of PAHs and metals are not conclusive. Depending on the combination of metals and PAHs, the exposure medium, the test organism, test duration and the studied endpoint, results range from additive, independent or antagonistic to synergistic mixture effects (e.g. Gust 2006; Shen et al. 2006). As shown for nickel and chlorpyrifos (Broerse and Van Gestel 2010a, b) toxicity is a dynamic process and mixture interactions may change over time. The presence of Cd may enhance pyrene toxicity by increasing accumulation. In addition, accumulation of the OH-pyrene metabolite was faster in the presence of Cd, probably because further metabolization was stagnated, resulting in a prolonged half life of OH-pyrene. Inhibition of metabolism has been shown to increase the bioconcentration of a variety of organic chemicals (Barron 1990). The mechanism behind interaction between Cd and pyrene might lie in the fact that both chemicals create oxidative stress. PAHs are known for enhancing the production of reactive oxygen species (ROS) like hydrogen peroxide (Gastaldi et al. 2007). Although Cd is not a redox metal (Satarug et al. 2003), it is believed to cause oxidative stress through the Fenton reaction producing radical species that might initiate lipid peroxidation (Banni et al. 2009). This was confirmed by a microarray study, showing the up regulation of transcripts encoding for monooxygenase and short-chain dehydrogenase (Nota et al. 2009). Cd can be sequestered by reduced glutathione (GSH) and/or metallothionein to prevent its adverse interaction with biomolecules (Wang et al. 2009). GSH and other thiols play a critical role in scavenging ROS. When exposed to both PAHs and Cd, GSH and other thiols might deplete, indirectly elevating ROS levels (Martelli and Moulis 2004; cited by Wang et al. 2009). Cd may also delay further metabolization of the phase 1 products of pyrene, by a depletion of possible conjugates. In the second phase, reactive phase 1 metabolites are conjugated with chemicals like glutathione or glucuronic acid (Baird et al. 2005; Jakoby and Ziegler 1990), which also may be involved in scavenging and detoxifying free Cd. The observed delay in further metabolization of OH-pyrene in the presence of Cd might be due to this.

We conclude that pyrene accumulation and metabolization in the collembolan *F. candida* was significantly influenced by the presence of cadmium. Since toxicity is a dynamic process, mixture effects may be dependent on exposure time. Further research is needed to underpin the actual mechanisms responsible for our findings and the possible consequences for toxicity.
